# An efficient feeder-free and chemically-defined expansion strategy for highly purified natural killer cells derived from human cord blood

**DOI:** 10.1016/j.reth.2023.05.006

**Published:** 2023-06-01

**Authors:** Tsutomu Nakazawa, Ryosuke Maeoka, Takayuki Morimoto, Ryosuke Matsuda, Mitsutoshi Nakamura, Fumihiko Nishimura, Shuichi Yamada, Ichiro Nakagawa, Young-Soo Park, Toshihiro Ito, Hiroyuki Nakase, Takahiro Tsujimura

**Affiliations:** a Grandsoul Research Institute for Immunology, Inc., Uda, Nara, 633-2221, Japan; b Clinic Grandsoul Nara, Matsui 8-1, Uda, Nara, 633-2221, Japan; c Department of Neurosurgery, Nara Medical University, Kashihara, Nara, 634-8522, Japan; d Department of Immunology, Nara Medical University, Kashihara, Nara, 634-8522, Japan

**Keywords:** Cord blood, Allogeneic NKC, Cell-based immunotherapy, Cancer immunotherapy, Glioblastoma

## Abstract

**Introduction:**

Natural killer cells (NKCs) are immune cells that can attack cancer cells through the direct recognition of ligands without prior sensitization. Cord blood-derived NKCs (CBNKCs) represent a promising tool for allogenic NKC-based cancer immunotherapy. Efficient NKC expansion and decreased T cell inclusion are crucial for the success of allogeneic NKC-based immunotherapy without inducing graft-versus-host reactions. We previously established an efficient ex vivo expansion system consisting of highly purified-NKCs derived from human peripheral blood. Herein, we evaluated the performance of the NKC expansion system using CB and characterized the expanded populations.

**Methods:**

Frozen CB mononuclear cells (CBMCs), with T cells removed, were cultured with recombinant human interleukin (rhIL)-18 and rhIL-2 under conditions where anti-NKp46 and anti-CD16 antibodies were immobilized. Following 7, 14, and 21 days of expansion, the purity, fold-expansion rates of NKCs, and the expression levels of NK activating and inhibitory receptors were assessed. The ability of these NKCs to inhibit the growth of T98G, a glioblastoma (GBM) cell line sensitive to NK activity, was also examined.

**Results:**

All expanded T cell-depleted CBMCs were included in over 80%, 98%, and 99% of CD3^−^CD56^+^ NKCs at 7, 14, and 21 days of expansion, respectively. The NK activating receptors LFA-1, NKG2D, DNAM-1, NKp30, NKp44, NKp46, FcγRIII and NK inhibitory receptors TIM-3, TIGIT, TACTILE, NKG2A were expressed on the expanded-CBNKCs. Two out of three of the expanded-CBNKCs weakly expressed PD-1, yet gradually expressed PD-1 according to expansion period. One of the three expanded CBNKCs almost lacked PD-1 expression during the expansion period. LAG-3 expression was variable among donors, and no consistent changes were identified during the expansion period. All of the expanded CBNKCs elicited distinct cytotoxicity-mediated growth inhibition on T98G cells. The level of cytotoxicity was gradually decreased based on the prolonged expansion period.

**Conclusions:**

Our established feeder-free expansion system yielded large scale highly purified and cytotoxic NKCs derived from human CB. The system provides a stable supply of clinical grade off-the-shelf NKCs and may be feasible for allogeneic NKC-based immunotherapy for cancers, including GBM.

## 1 Introduction

Natural killer cells (NKCs) represent a crucial component of the human immune system, representing a front line defense against tumor-forming cells and pathogens. The natural killing activity of peripheral blood (PB) lymphocytes against aberrant cells, including, the discovery of tumor- or virus-infected cells in both mice and humans, dates back to the 1970s [[1], [2], [3]]. NKCs are crucial for tumor immunosurveillance due to their unique capability of identifying and attacking abnormal cells without requiring prior sensitization.

The “missing self” hypothesis, put forth in 1981 by Kärre and Ljunggren, postulated that natural killer cells (NKCs) target cells that do not display adequate levels of self-major histocompatibility complex (MHC) class I molecules belonging to the host [4,5]. This concept emerged from studies investigating the role of MHC molecules in the NKC response to cancer cells and has served as a foundational framework for understanding target cell recognition by NKCs for over 15 years. The hypothesis gained widespread recognition when it was demonstrated that NKCs also attack normal cell types, such as bone marrow cells, in the absence of MHC class I molecules [6]. In humans, MHC class I molecules are referred to as human leukocyte antigen (HLA) class I. More precisely, NKCs kill abnormal cells when they do not express sufficient levels of MHC- molecules and express molecules recognized by activating NK receptors.

The molecular mechanisms behind this function have been studied extensively, with crucial surface receptors, including NK activating and inhibitory receptors, being subsequently characterized. For instance, NK activating receptors include lymphocyte function-associated antigen (LFA-1), NK group 2D (NKG2D), DNAX accessory molecule-1 (DNAM-1), NKC p30-related protein (NKp30), NKp44, NKp46, and Fc fragment of IgG receptor III (FcγRIII). Inhibitory receptors consist of programmed death (PD)-1, lymphocyte activation gene (LAG)-3, T cell immunoglobulin mucin family member (TIM)-3, T cell immunoreceptor with immunoglobulin and ITIM domains (TIGIT), T cell activation, increased late expression (TACTILE), NKG2A, and certain killer Ig-like receptors (KIRs), such as KIR2DL [[7], [8], [9], [10]]. Inhibitory KIRs recognize a lack of MHC or HLA class I expression and are involved in the “missing hypothesis” [11]. NKC activation and function hinge on the combination of signals stemming from both activating and inhibitory receptors, facilitating host defense against atypical cells and circumventing adverse autoimmune responses. Furthermore, a decrease in surface MHC or HLA along with a simultaneous rise in stress ligands recognized by NKC receptors on tumor cells can lead to increased activation of NKCs. The elimination of target cells is achieved either directly through cytotoxic pathways or indirectly through cytokine secretion. This elimination process is executed via the release of lytic granules containing perforin and granzymes or by triggering death receptor-mediated apoptosis through the interaction of Fas ligand or tumor necrosis factor-related apoptosis-inducing ligand (TRAIL) [9,12].

NKCs have garnered attention as a promising alternative platform for T cell-based immunotherapy due to their highly cytotoxic and T cell receptor/HLA-unrestricted effector function. NKC-based immunotherapy involves the unique recognition of cancer cells via mechanisms that differ from that of T cell-based immunotherapy and are potentially effective against cancers. Moreover, early clinical trials investigated the adoptive transfer of ex vivo expanded autologous NKCs as a treatment for various types of cancer, including renal cell carcinoma [13], lymphoma [14,15], breast cancer [15,16], digestive cancer [17], colon, and lung cancers [18]. Despite low toxicity and positive treatment reception, the anti-tumor effect was observed to be limited in patients with these disease. The main obstacle encountered in the adoptive transfer of NKCs was the functional inhibition caused by self-recognition via inhibitory KIRs present on the NKCs, which match the presence of HLA class I on tumor cells. This recognition resulted in a blockade of the activation process [7]. Another limiting factor was that the patients had undergone extensive pre-treatment (e.g., antitumor reagents or steroids) prior to NKC collection and therapy, which had a negative impact on ex vivo expansion and in vivo functions of the NKCs after infusion [19]. To resolve these limitations, the use of ex vivo activated allogeneic NKCs was explored.

Various sources of human NKCs, such as bone marrow (BM) and cord blood mononuclear cells (CBMCs), pluripotent stem cells (iPSCs), and embryonic stem cells (ESCs), have been identified. Additionally, peripheral blood mononuclear cells (PBMCs) are considered potential candidates for generating allogeneic NKCs in vitro [[20], [21], [22], [23]]. While ESC- and iPSC-derived NKCs show potential, they face challenges such as limited induction efficiency and a lengthy induction process (approximately 1–2 months) [24,25]. Moreover, the culturing process is expensive due to the substantial amounts of cytokines and defined serum-free medium required for induction. However, there are similar concerns for both iPS and ES cells, as well as CD34 positive BM- and CB hematopoietic stem/progenitor cell-derived NKCs. The direct expansion approach for NKCs utilizing PBMCs and CBMCs can address the limitations of stem cell-derived NKCs and may serve as potential candidates for off-the-shelf allogeneic NKC therapies. NKCs represent about 10% of all lymphocytes in PB, while in CB, they constitute up to 30% of the lymphocytes [26,27]. It has been reported that CB does not have the negative impact of heavy pre-treatment prior to the expansion of NKCs [21]. Thus, CB could be a prominent and promising source of therapeutic effector NKCs compared to PB.

We have previously established an effective ex vivo expansion system for highly purified NKCs derived from human PB, employing a combination of cytokines and antibodies targeting NK receptors in a feeder-free environment [28,29]. Moreover, its anti-tumor effect in allogeneic glioblastoma (GBM) was also reported for clinical application [28,30,31]. In this study, we evaluated and monitored the performance of the NKC expansion system using CB and characterizing the associated phenotype. These findings demonstrate that our system can consistently yield large scale highly purified and cytotoxic NKCs, which can be used for allogeneic NKC-based immunotherapy for treating cancer, including GBM.

## 2 Materials and methods

### 2.1 Human CB mononuclear cells

CBMCs were acquired from RIKEN BioResource Research Center (RIKEN BRC; Tsukuba, Ibaraki, Japan) following approval of the Nara Medical University Ethics Committee (Approval number 3310) and conducted its applicable guidelines.

### 2.2 Antibody-coated plate

Anti-human NKp46 antibodies (clone 195,314, R&D Systems, Minneapolis, MN, USA) and anti-human CD16 antibodies (clone 3G8, Thermo Fisher Scientific, Waltham, MA, USA) (both 5 μg/mL) were prepared in phosphate-buffered saline (PBS; Kohjin Bio, Saitama, Japan) containing 0.1% human serum albumin (FUJIFILM Wako Pure Chemical, Tokyo, Japan). A 1.5 or 0.7 mL antibody solution was transferred to 24- or 12 well-well plates (Corning, Steuben, NY, USA) and incubated at 4 °C for >12 h for antibody immobilization. The antibody solution was then removed from the flask, the flask was washed with PBS, and subsequently used for human NKC culturing.

### 2.3 NKC expansion

The specific approach for human NKC expansion was carried out as previously outlined [29]. In brief, frozen CBMCs were sourced from RIKEN BRC derived from three volunteers (0 years old, 1 male, and 2 females). The CD3 fraction of the CBMCs was depleted using a RosetteSep™ Human CD3 Depletion Cocktail (STEMCELL Technologies, Vancouver, Canada). A total of 2 × 10^6^ or 1 × 10^6^ of CD3-depleted cells were placed in 6- or 12-well anti-NKp46 and antiCD16 antibody immobilization plates containing AIM-V medium (Thermo Fisher Scientific) supplemented with 10% autologous plasma, 50 ng/mL recombinant human IL-18 (rhIL-18, Medical & Biological Laboratories, Nagoya, Japan), and 3000 IU/mL rhIL-2 (COREFRONT, Tokyo, Japan) at 37 °C in a humidified incubator containing 5% CO_2_. AIM-V medium supplemented with 3000 IU/mL rhIL-2 was replenished as needed for 28 days. The expanded cells were frozen in CELLBANKER 2 (Nippon Zenyaku Kogyo Co., Ltd., Fukushima, Japan). To assess the inhibitory effects on tumor cell growth, frozen cells were revived in AIM-V medium supplemented with 10% heat-inactivated fetal bovine serum (FBS; MP Biomedicals, Tokyo, Japan) and 3000 IU/mL rIL-2, followed by a two-day incubation.

### 2.4 Human cell line

In this study, a standard human glioblastoma (GBM) cell line, T98G (RIKEN BRC), known for its sensitivity to NK activity [28], was utilized. The cells were cultured in Dulbecco's modified Eagle's medium (Thermo Fisher Scientific) enriched with 10% heat-inactivated FBS, 100 U/mL penicillin, and 100 mg/mL streptomycin (Thermo Fisher Scientific) at 37 °C in a humidified environment containing 5%CO_2_.

### 2.5 Flow cytometry

The cells were stained with the appropriate antibodies and fixed in 1% paraformaldehyde containing PBS (FUJIFILM Wako Chemicals) at 4 °C for 30 min. Data were obtained using Spectral Cell Analyzer SA3800 (SONY, Tokyo, Japan) and BD FACSCalibur. Data were analyzed using SA3800 and FlowJo v10 (BD Biosciences). The respective antibodies used for flow cytometry are listed in the supplemental materials and methods.

### 2.6 Cytotoxicity-mediated real time cell growth inhibition assays

The inhibition of growth in the NK activity-sensitive GBM cell line, T98G, by expanded NKC-containing populations was examined using xCELLigence RTCA DP (real-time cell analysis dual purpose) instruments (ACEA Biosciences, San Diego, CA, USA), as previously detailed [29,30,32]. In brief, 100 μL of complete medium was added to each well on an E-plate 16 (ACEA Biosciences). Background impedance was measured at 37 °C in a humidified environment with 5% CO2. T98G cells [2 × 104/well (50 μL)] were seeded into each well as target (T) cells and cultured for 20 h. The expanded NKC-containing populations (50 μL) were introduced to each well as effector (E) cells at E:T ratios of 0.5:1 and 1:1. Impedance measurements were recorded every 5 min for 6 h. Data analysis was performed using the RTCA Software Package 1.2 (ACEA Biosciences). Cytotoxicity, calculated from impedance values (cell index), was previously reported [33] and slightly modified. The following formula was used: (1 − normalized cell index of target cells co-cultured with each sample ÷ normalized cell index of target cells) × 100 (%).

### 2.7 Statistical analysis

Statistical evaluations were conducted with Prism 8 (GraphPad Software Inc., San Diego, CA, USA). Data were presented as the mean ± standard error (SE) or standard deviation (SD). To determine the statistical significance of differences between groups, a two-way analysis of variance (ANOVA) followed by Tukey's test was employed. A P-value of <0.05 was deemed statistically significant.

## 3 Results

### 3.1 Ex vivo expansion of human CBNKCs stimulated by NKC activating receptor antibodies and defined cytokines

**Fig. 1:**
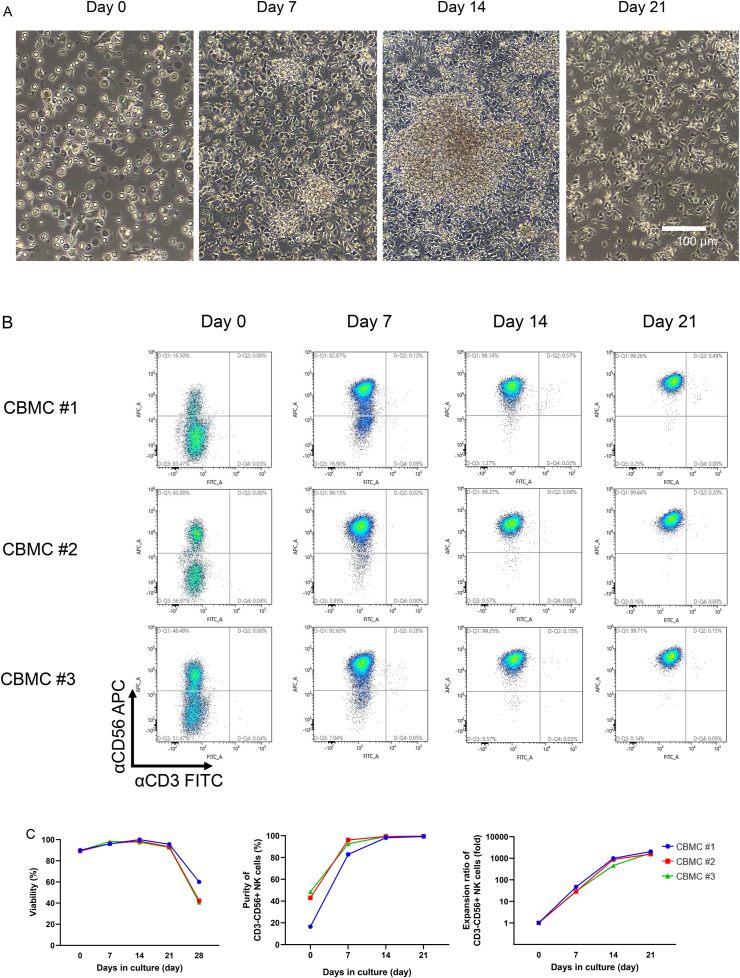
Ex vivo expansion of human CBNKCs stimulated by NK activating receptor antibodies and defined cytokines. (a) Representative morphological patterns of the expanded cells on days 0, 7, 14, and 21. (b) The cellular proportion (CD3^−^CD56-: non-T, non-NKC, CD3^−^CD56^+^: NKC, CD3^+^CD56-: T cells; and CD3^+^CD56^+^: NKT cells) was analyzed by flow cytometry. The dot plots on days 0, 7, 14, and 21 of three donors (CBMC#1, #2, and #3) are depicted. The x-axis and y-axis show the fluorescence intensity of cells stained with FITC-conjugated anti-CD3 and APC-conjugated anti-CD56 antibodies, respectively. (c) The kinetics of the total cell viability (left), NKC purity (center), and NKC expansion ratio (right) during the expansion period. Blue lines and circles, red lines and squares, green lines and triangles indicate CBMC#1, #2, and #3, respectively.

To assess the effectiveness of the specific culture conditions required for NKC expansion, T cell-depleted CBMCs from three donors were cultured ex vivo. Following T cell depletion, approximately 2 × 10^6^ cells were gathered from 10^7^ frozen-CBMCs, obtaining an average of 36% CD3^−^CD56^+^ NKCs. Donors 1, 2, and 3 produced 16.5%, 43.0%, and 48.5% CD3CD56+ NKCs, respectively, with CD3^+^ cells comprising 0.03%, 0.04%, and 0.04%, respectively (Table 1). Donor-derived T cell-depleted PBMCs were expanded using a combination of cytokines, rhIL-18 and high-dose rhIL-2, on anti-NKp46 and/or anti-CD16 antibody immobilization plates at a cell density of 106 cells/mL in 1 mL of culture medium. Cell viability was evaluated on days 0, 7, 14, 21, and 28. Total and CD3^−^CD56^+^ NKC numbers were calculated on days 0, 7, 14, and 21. Fig. 1a displays the representative morphological patterns of the expanded cells on days 0, 7, 14, and 21. On day 7, the expanded cells formed clusters and underwent proliferation. On day 14, the expanded cells created larger clusters compared to day 7 and exhibited explosive proliferation. By day 21, the cell count had increased, while the number of cell clusters had decreased. Additionally, there was a rise in the number of cells adhering to the plastic surface.

**Table 1:** Summary of NKC purity and expansion ratio in the expanded NKC-containing populations. CD3^−^CD56^+^ NKC numbers were calculated based on the cell number and percentage of CD3^−^CD56^+^ cells. The expansion ratios were determined by each culture day of the CD3^−^CD56^+^ NKC number divided by the day 0 CD3^−^CD56^+^ NKC number. N.D. indicates not determined.

	Sample	Day 0	Day 7	Day 14	Day 21	Day 28
Expanded cell viability (%)	CBMC#1	90.0	96.0	100	95.6	60.0
	CBMC#2	89.2	96.2	98.6	93.2	42.3
	CBMC#3	89.5	98.3	97.5	92.6	40.6
Frequency of CD3^−^CD56^+^NK cells (%)	CBMC#1	16.5	82.9	98.1	99.3	N.D.
	CBMC#2	43.0	96.1	99.4	99.6	N.D.
	CBMC#3	48.5	92.6	99.3	99.7	N.D.
Expansion ratio of CD3^−^CD56^+^NK cells (fold)	CBMC#1	–	47.1	993.3	2066.7	N.D.
	CBMC#2	–	28.7	879.0	1560.4	N.D.
	CBMC#3	–	29.4	463.0	1755.6	N.D.

The cell viability of all expanded cells was >90% on days 7,14, and 21, but had substantially decreased to less to 60% by day 28 (Fig. 1C left panel). The purity of CD3^−^CD56^+^ NKCs of donors 1, 2, and 3 were 82.9%, 96.1%, and 92.6% on day 7, and 98.1%, 99.4%, and 99.3% on day 14, and 99.3%, 99.6%, and 99.7% on day 21, respectively. On day 7, the NKC purity was over 80%, although it varied among the samples. The purity was over 98% on day 14 and reached almost 100% on day 21 in all of the tested samples (Fig. 1b and c middle panel). The NKC expansion ratios of donors 1, 2, and 3 were 47.1-, 28.7-, and 29.4-fold on day 7; 993.3-, 879.0-, and 463.0-fold on day 14; and 2066.7-, 1560.4-, 1755.6-fold on day 21, respectively (Fig. 1C right panel). The data are summarized in Table 1. Our specific NKC expansion system stably and efficiently yielded a high purity of NKCs by 14 days (NKC purity: >98%, NKC expansion ratio: 778.4 ± 161.9-fold expansion; average ± SE).

### 3.2 Monitoring of NK activation and inhibitory receptor expression on expanded CBNKCs

The expression of the NK activation receptors, LFA-1, NKG2D, DNAM-1, NKp30, NKp44, NKp46, and FcγRIII, as well as NK inhibitory receptors, PD-1, LAG-3, TIM-3, TIGIT, TACTILE, NKG2A, and KIR on the expanded CBNKs was determined on days 7, 14, and 21 of the culture process.

**Fig. 2:**
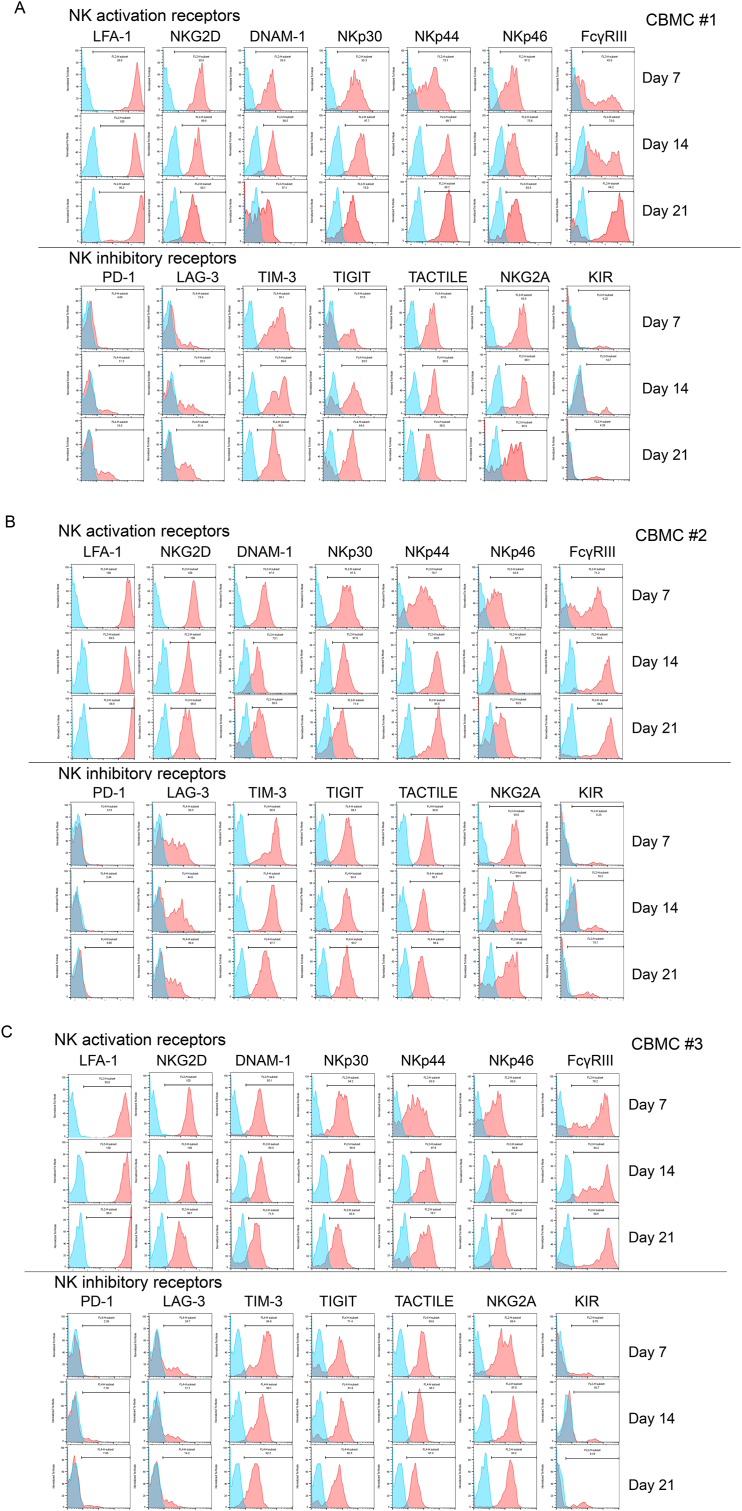
NK activating and inhibitory receptor expression on the expanded CBNKCs. The NK activation receptors, LFA-1, NKG2D, DNAM-1, NKp30, NKp44, NKp46, FcγRIII, and NK inhibitory receptors, PD-1, LAG-3, TIM-3, TIGIT, TACTILE, NKG2A, and KIR were evaluated by flow cytometry. Histogram data of each receptor on the expanded CBNKCs gated on the CD56 positive population. Days 7, 14, and 21 are arranged to upper, middle, and lower panels in the NK activating (tops) and inhibitory receptor (bottoms) regions. (a), (b), (c) indicate CBMC#1, #2, and #3, respectively.

**Fig. 3:**
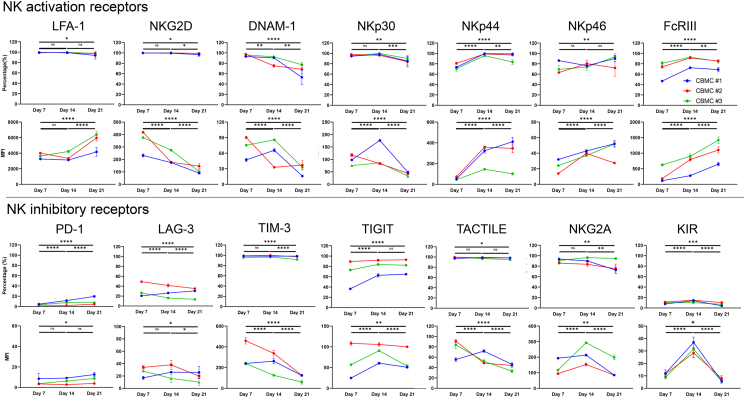
Kinetics of NK activating and inhibitory receptor expression on the expanded CBNKCs in the expansion process. The frequency and mean fluorescent intensity (MFI) of NK activating and inhibitory receptors on day 7, 14, and 21 are shown. Blue lines and circles, red lines and squares, green lines and triangles indicate CBMC#1, #2, and #3, respectively. Data are expressed as the mean ± SE of triplicate experiments. Statistical differences were determined by a two-way ANOVA followed by a Tukey's test. ∗∗∗∗*P* < 0.0001; ∗∗∗*P* < 0.001; ∗∗*P* < 0.01; ∗*P* < 0.05, ns: not signiﬁcant.

In donor 1, the expression of LFA-1, NKG2D, DNAM-1, NKp30, NKp44, NKp46, and FcγRIII NK activating receptors and TIM-3, TIGIT, TACTILE, and NKG2A NK inhibitory receptors were universally expressed in the expanded CBNKs on all days tested; however, the expression varied depending on the expansion period. Among the NK activating receptors, LFA-1-positive cells and the MFI remained unchanged on days 7 and 14. While the LFA-1-positive cells were somewhat decreased on day 21, LFA-1 expression was upregulated on day 21. NKG2D-positive cells were somewhat decreased in a culture day-dependent manner. The MFI was gradually decreased in a culture day-dependent manner. DNAM-1-positive cells were decreased in a culture day-dependent manner and the MFI was up-regulated on day 14 but down-regulated on day 21. NKp30-positive cells and the MFI were up-regulated on day 14 but down-regulated on day 21. NKp44-positive cells were increased on day 14 and remained elevated at day 21, respectively. The MFI was gradually up-regulated in a culture day-dependent manner. NKp46-positive cells were decreased on day 14 but increased on day 21. The MFI was up-regulated in a culture day-dependent manner. FcγRIII-positive cells were increased on day 14 and remained elevated on day 21. The MFI was up-regulated in a culture day-dependent manner. Of the NK inhibitory receptors, both the PD-1-positive cells and MFI were low in all days tested. PD-1-positive cells and the MFI were gradually increased in a culture day-dependent manner. LAG-3-positive cells and MFI were intermediate on all days tested. LAG-3-positive cells increased; however, MFI expression was slightly up-regulated according in a culture day-dependent manner. TIM-3-positive cells and MFI were high on all days tested. TIM-3-positive cells remained largely unchanged but were down-regulated on day 21. TIGIT-positive cells were increased on day 14 and remained elevated on day 21. The MFI was also up-regulated on day 14 but was slightly down regulated on day 21. TACTILE-positive cells remained consistent for all days tested. The MFI was up-regulated on day 14 but down-regulated on day 21. The number of NKG2A-positive cells gradually decreased in a culture day-dependent manner. The MFI was moderately increased on day 14 but decreased on day 21. The number of KIR-positive cells and MFI were very low, but were upregulated on day 14 and downregulated on day 21 (Fig. 2, Fig. 3).

In donor 2, similar donor 1, LFA-1, NKG2D, DNAM-1, NKp30, NKp44, NKp46, FcγRIII, TIM-3, TIGIT, TACTILE, and NKG2A were universally expressed in the expanded CBNKs on all days tested, although the expression varied depending on the expansion period. Among the NK activating receptors, The LFA-1-positive cells were somewhat decreased on day 21; however, the MFI was downregulated on day 14 and upregulated on day 21. NKG2D-positive cells remained consistent on all days tested. The MFI gradually decreased in a culture day-dependent manner. The number of DNAM-1-positive cells were decreased in a culture day-dependent manner and the MFI was down-regulated on day 14 and remained consistent on day 21. The number of NKp30-positive cells was slightly decreased on day 21 and the MFI was down-regulated in a culture day-dependent manner. NKp44-positive cells were increased and kept on days 14 and 21, respectively. The MFI were gradually up-regulated in a culture day-dependent manner. NKp46-positive cells were increased on day 14 and kept in day 21. The MFI was up-regulated on day 14 and down-regulated on day 21. FcγRIII-positive cells were increased on day 14 and decreased on day 21. The MFI was up-regulated in a culture day-dependent manner. Among the NK inhibitory receptors, PD-1-positive cells and MFI exhibited low expression and remained constant on all days tested. LAG-3-positive cells decreased in a culture day-dependent manner. The MFI was down-regulated on day 21. TIM-3-positive cells remained largely unchanged on all days tested. The MFI was down-regulated according to culture period. TIGIT-positive cells and the MFI were remained constant on all days tested. TACTILE-positive cells remained constant on all days tested. The MFI was down-regulated on day 14 and kept on day 21. NKG2A-positive cells slightly and gradually decreased in a culture day-dependent manner. The MFI was up-regulated on day 14 but decreased on day 21. KIR-positive cells and the MFI levels were initially very low, were upregulated on day 14, and downregulated on day 21 (Fig. 2, Fig. 3).

In donor 3, similar to donors 1 and 2, LFA-1, NKG2D, DNAM-1, NKp30, NKp44, NKp46, and FcγRIII NK activating receptors, as well as TIM-3, TIGIT, TACTILE, and NKG2A NK inhibitory receptors were universally expressed in the expanded CBNKs on all days tested; however, the expression varied depending on the expansion period. Among the NK activating receptors, LFA-1-positive cells were kept in all days tested and the MFI gradually increased according to culture period. NKG2D-positive cells remained consistent on all days tested. The MFI was gradually decreased in a culture day-dependent manner. DNAM-1-positive cells were decreased according to the culture day. The MFI was up-regulated on day 14 and down-regulated on day 21. NKp30-positive cells were slightly increased on day 14 and decreased on day 21. The MFI was up-regulated on day 14 and down-regulated on day 21 in a culture day-dependent manner. NKp44-positive cells were increased and decreased on days 14 and 21, respectively. The MFI was up-regulated on day 14 and down-regulated on day 21. NKp46-positive cells in a culture day-dependent manner. The number of FcγRIII-positive cells were increased on day 14 and decreased on day 21. The MFI was up-regulated in a culture day-dependent manner. Among the NK inhibitory receptors, the PD-1-positive cells and MFI were low. PD-1-expressing cells were increased on day 14 and remained consistent on day 21. The MFI was up-regulated and down-regulated on days 14 and 21, respectively. The number of LAG-3-positive cells and MFI were decreased and down-regulated, respectively, according to the culture period. TIM-3-positive cells were increased on day 14 and kept in day 21. The MFI was down-regulated in a culture day-dependent manner. TIGIT-positive cells were increased in day 14 and kept in day 21. The MFI was up-regulated on day 14 and down-regulated on day 21. TACTILE-positive cells gradually decreased in a culture day-dependent manner. The MFI was gradually down-regulated on day 14 and day 21. NKG2A-positive cells were slightly increased on day 14 and remained consistent until day 21. The MFI was up-regulated on day 14 but down-regulated on day 21. The number of KIR-positive cells were increased on day 14 and decreased on day 21. The MFI was up-regulated on day 14 and down-regulated on day 21 (Fig. 2, Fig. 3).

The combined receptor expression data from the donors revealed that NKG2D was consistently and significantly down-regulated in a culture day-dependent manner. Conversely, FcγRIII were up-regulated in a culture day-dependent manner. KIR was up-regulated on day 14 and down-regulated on day 21 (Fig. 3). Overall, LFA-1, NKG2D, DNAM-1, NKp30, NKp44, NKp46, and FcγRIII, as well as the NK inhibitory receptors TIM-3, TIGIT, TACTILE, and NKG2A, were universally expressed in the expanded CBNKCs of all tested donors; however, the expression varied in a time-dependent manner. Furthermore, two out of the three expanded-CBNKCs weakly expressed PD-1, and one out of three exhibited minimal PD-1 expression. LAG-3 expression differed among donors, and consistent changes could not be identified based on the expansion period.

### 3.3 Evaluation of the cytotoxicity-mediated growth inhibition of an NK activity-sensitive GBM cell line by the expanded CBNKC-containing populations

**Fig. 4:**
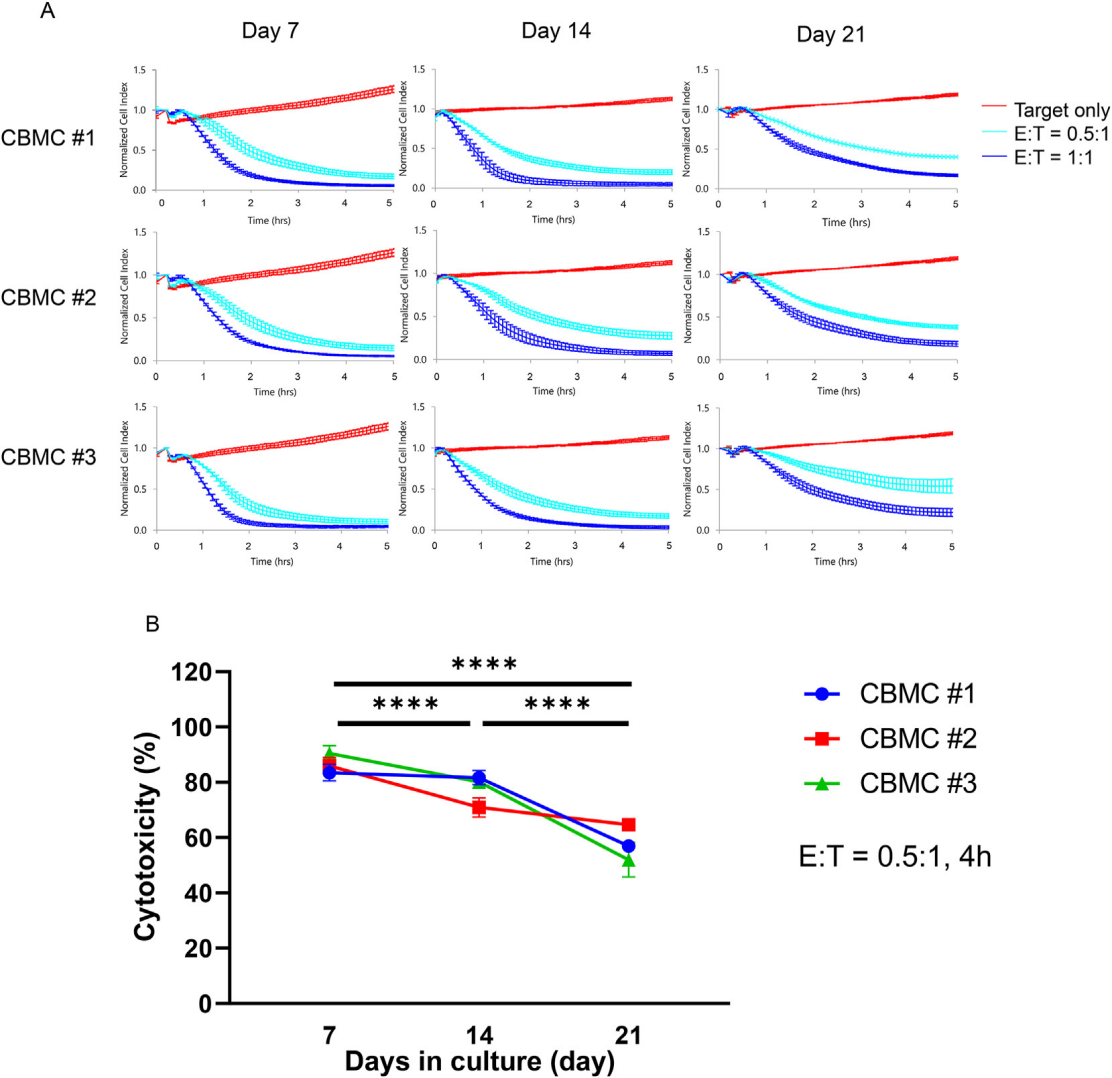
Cytotoxicity-mediated growth inhibition effects of T98G GBM cells by the expanded CBNKC-including populations. (a) Images depict the real-time growth inhibition of a NK activity-sensitive GBM cell line, T98G, by the expanded CBNKC-included population. The left, middle, and right panel lines depict the 7-, 14-, 21-day cultures, respectively. The top, intermediate, and bottom lines depict CBMC#1, #2, and #3, respectively. The X- and Y-axes respectively depict the co-culture time and relative normalized cell index of each time point divided by the cell index of the co-culture starting point. Data represent the mean ± SD of triplicate experiments. The red, light blue, and blue lines indicate target (T98G) only; the effector (the expanded CBNKC-including population) to target (E:T) ratio was 0.5:1 and 1:1, respectively. (b) The cytotoxicity of the expanded CBNKC-including population on T98G cells. The X- and Y-axes respectively depict culture days for the CBNKC-including population and cytotoxicity. Blue lines and circles, red lines and squares, and green lines and triangles indicate CBMC#1, #2, and #3, respectively. Data represent the mean ± SE of triplicate experiments. Statistical differences of the integrated data of three donors were determined by a two-way ANOVA followed by a Tukey's test. ∗∗∗∗*P* < 0.0001.

The growth-inhibiting effects of cytotoxicity mediated by the expanded cell populations on the standard T98G human GBM cell line (an NK-sensitive cell line) were assessed using an RTCA system. T98G cells were seeded into an E plate for a 20-h incubation. The retrieved expanded cells were introduced to each well of an E plate (16-well plates in the RTCA system) at an E:T ratio of 0.5:1 and 1:1. The recovered expanded cell population viability was >90%. After co-culturing with the NKC-containing population, a time-dependent reduction in cell numbers revealed the level of T98G cell growth inhibition by all expanded cell populations (Fig. 4a). The cytotoxicity at 4 h was determined using the formula specified in the materials and methods section. The NKC-containing population showed distinct and strong cytotoxicity, albeit slightly varied among donors. The integration of cytotoxicity data on each day of culture revealed that the cytotoxicity of the NKC-containing populations was significantly and gradually decreased in a culture period-dependent manner (Fig. 4b). Although the NKC purity of the expanded populations on day 7 was low compared to that on days 14 and 21 (Table 1), the cytotoxicity on day 7 was high compared to that on days 14 and 21 (Fig. 4b).

## 4 Discussion

To our knowledge, this is the first report of our previously established NKC expansion system that is able to be adapted to both human PBNKCs and CBNKCs. The expanded CBNKCs included highly-purified and cytotoxic NKCs, with low T cell contamination from 7 days to 21 days of culture. The results suggest that the NKC expansion system has the potential to be implemented in clinical research and enable successful allogeneic NKC-based immunotherapy for cancers without the risk of graft-versus-host disease (GVHD). Despite the fact that allogeneic NKC therapy can produce strong antitumor effects via graft-versus-leukemia (GVL) mechanisms, there is a potential for GVHD [34]. According to other studies, around 7% of patients who received allogeneic NKC therapy with KIR ligand-mismatch experienced GVHD. The increased incidence of GVHD was observed in patients with higher CD3 chimerism, indicating the presence of mixed T cells that could trigger a GVHD response [35,36]. Thus, an enhanced purity of NKCs can reduce the incidence of GVHD. The present study showed that CBMCs expanded by our system included 0.05–0.09% and 0.00–0.03% of T cells at day 7 and 14, respectively. Using the same method to culture PBMCs, 0.22–3.5% of T cells were observed at day 14 [29]. We considered that a reason for the low T cell contamination in the expanded-CBNKCs was that T cells in CB had not exposed to specific antigens, which were almost virgin T cells. Neonatal CB-derived T cells are antigenically naive and constitute an unprimed cell population. Conversely, T cells derived from adult PB have experienced antigenic exposure and were utilized as a source of in vivo primed cells exhibiting acquired “memory” [37]. Therefore, it is possible that the T cells in CB are less responsive to IL-2 stimulation and would display lower amplification in a IL-2-based culture system. Additional cases and detailed analysis are required to confirm this point.

The present study demonstrated that the expanded-CBNKCs expressed NK activating and inhibitory receptors. The expression of NKG2D was consistently decreased over a prolonged culture period in three donors. Moreover, the frequency of PD-1 expression was low and there were no consistent changes in expression of other inhibitory receptors, including LAG-3, TIM-3, TIGIT, TACTILE, and CD94. On the other hand, the cytotoxicity of NKC-including populations was significantly and gradually decreased in a culture period-dependent manner (Fig. 4B). These accumulated data indicate that there was a decrease in cytotoxicity based on the expansion period. This change may be related to NKG2D expression and was not likely due to changes in the expression of the tested inhibitory receptors. In addition, NKC exhaustion may be related to decreased NKC cytotoxicity. NKC, B cell, and T cell exhaustion can be defined by impaired function caused by antigenic overstimulation [38]. Both NKCs and T cells exhibit reduced effector functions and phenotypic changes. However, unlike T cell exhaustion, there is no clear consensus on the definition of NKC exhaustion [39]. Mitochondrial metabolic pathways play an important role in regulating T cell fate, function, and longevity in T cell exhaustion [40]. Thus, it is possible that a similar process is occurring in expanded CBNKCs.

It has previously been reported that CBNKCs were expanded by co-culturing with the gene-modified K562 leukemia cell line [41,42]. Although commonly used in product development and early clinical stages, the use of feeder cells poses limitations in advanced manufacturing processes. Despite growth arrest by γ-irradiation, residual contamination of feeder-derived impurities may remain in the final product. Therefore, specific release criteria for feeder-cultured products should be established. One approach to address concerns with the use of feeder cells is to label them with a fluorescent tag or express a suicide gene. Additionally, the use of feeder cells in CBNKC expansion carries a risk of viral or bacterial infection, including mycoplasma. Therefore, a feeder-free method is preferable to ensure safety and enhance the control and robustness of the manufacturing process [43,44]. Mu et al. reported that they used OK432, a preparation of heat-killed *Streptococcus pyogenes*, as stimulation [45]. The expansion ratio of our system vs the system reported by Mu et al. on days 7, 14, and 21 were 29.4–47.1-fold vs approximately 10 to 90-fold, 463.0–993.3-fold vs approximately 100–500, 1560.4–2066.7-fold vs approximately 500-1500-fold, respectively. The NKC purity of our system compared to the Mu et al. system on days 7, 14, and 21 were 82.9%–96.1% vs approximately 40%–60%, 98.1%–99.4% vs 60%–80%, and 99.3%–99.4% vs approximately 88%–92%, respectively. Our system demonstrated a stable expansion system without donor differences and a high expansion efficiency on days 14 and 21 and high purity in all tested cells. Furthermore, determining sterility in this system is challenging due to the presence of OK432, making it hard to differentiate between viable and dead bacteria during culture. Overall, our NKC expansion system may present the optimal approach for allogeneic NKC-based immunotherapy.

Autologous infusions of NKCs constitute the initial and primary target of NKC-based immunotherapy. The main advantage of this approach is the ease of using the patient's own blood as a cell source, eliminating the need for immunosuppressive therapy and reducing the risk of GVHD. Studies have shown that the infused cells can expand in vivo, but their response to hematological or solid cancers is modest. This may be due in part to inhibitory interactions between autologous NKCs and self HLA class I molecules, as reported in previous studies [16,46]. Furthermore, individuals who underwent these infusions have possibly been extensively pretreated before cell collection and therapy. This may have adversely impacted the expansion and function of NKCs according to a previous study [46]. In GBM, alkylating agents of cancer treatment, like temozolomide, commonly used in cancer treatment, usually restrict peripheral lymphocyte numbers and impede hematopoietic stem cell proliferation [47]. Additionally, steroids are frequently administered to manage brain edema in GBM patients after surgical intervention and concurrent chemoradiotherapy. The adverse consequences of glucocorticoids encompass lymphopenia, hyperglycemia, and susceptibility to infections due to their immunosuppressive effects [48]. Consequently, numerous groups have shifted their attention from autologous to allogeneic NKC therapies.

Allogeneic NKCs have been shown in various reports to have the potential to induce remission or prevent relapse in patients with hematological malignancies, such as acute myeloid leukemia (AML) and multiple myeloma (MM), through in vitro expansion and activation for hematopoietic stem cell transplantation or adoptive NKC-based immunotherapy [19,23]. In a partial report of a phase I trial, five out of nine patients with refractory acute myeloid leukemia (AML) exhibited complete remission. When two patients with transient significant leukemic cell loss were included, the response rate was 77.8% [49]. This result is similar to the initial trial data of CD19-targeted chimeric antigen receptor (CAR)-T cells have been approved in the United States for juvenile acute lymphoblastic leukemia [50]. Additionally, clinical trials have reported complete remission in elderly or poor prognosis individuals, as well as a 100% event-free survival rate at 18 months in a pediatric cohort treated with allogeneic NKCs for AML [51]. Allogeneic NKC-based immunotherapy has demonstrated clinical efficacy either as a standalone treatment or in combination with conventional therapies. Chu et al. provided a comprehensive review of clinical trials investigating the infusion of NKC in cancer patients, with ongoing studies demonstrating promising clinical potential for allogeneic NKC transfer [52]. Liang et al. reported that in recurrent breast cancer, allogeneic NKC-based immunotherapy had better clinical efficacy compared to autologous therapy, improving quality of life, reducing circulating tumor cells, and decreasing levels of carcinoembryonic antigen and cancer antigen 15-3 (CA15-3), while significantly enhances immune function [53]. In contrast, adoptive cell treatment with allogeneic NKCs derived from PB have similar disadvantages to that of autologous NKCs, including the rapid ex vivo expansion and activation of clinical-grade [52]. Our sophisticated NKC expansion system could stably and potently yield allogeneic NKCs derived from PB and CB. Additionally, our system could stably supply allogeneic NKCs in combination with the global CB bank, which is readily available. Thus, allogeneic CBNKCs obtained using our system could aid in the stable conduction of clinical research in both leukemias and solid tumors.

GBM, categorized as grade IV by the World Health Organization, represents the most common and aggressive form of primary brain tumor [54]. The standard treatment, with involves surgical resection, followed by chemotherapy and radiotherapy results in a median overall survival of merely 15–17 months [55]. This highlights the need for innovative treatment approaches for GBM patients, with immunotherapy emerging as a promising supplementary therapy. In our previous research, we showcased the antitumor effects of allogeneic PB-derived NKCs against GBM, both in vitro and in vivo, as well as against 3-dimensional GBM cell-derived spheroids [9,31]. Further investigation is required to determine the comparative anti-tumor efficacy of CBNKCs and PBNKCs against GBM, as well as to assess their clinical applicability.

We confirmed that our previously reported system used for PBNKC expansion can be applied to an efficient, large-scale, feeder-free expansion system for highly purified CBNKCs. Therefore, this expansion system provides a safe and stable supply of clinical grade off-the-shelf expanded-CBNKC that may be feasible for allogeneic NKC-based immunotherapy for the treatment of cancers, including GBM. The expression profile of various NK activating and inhibitory receptors on CBNKCs may be information of optimizing CBNKC-based immunotherapy.

## Ethics statement

CBMCs were acquired from RIKEN BioResource Research Center (RIKEN BRC; Tsukuba, Ibaraki, Japan) with the consent of the Nara Medical University Ethics Committee (Approval number 3310) and conducted by following its guidelines.

## Author contributions

Conceptualization, design, guidance, methodology, investigation, data analysis, and writing (original draft preparation, review, and editing): T.N. (Tsutomu Nakazawa) and R.Mae. (Ryosuke Maeoka); conceptualization and writing (review and editing): R.Mat. and T.T.; writing (review and editing): T.M., F.N., M.N., S.Y., Y.S., I.N., Y.-S.P., T.I. and H.N. All authors made important contributions to the experiments. All authors have read and agreed to the published version of the manuscript.

## Funding

None.

## Conflicts of interest

Tsutomu Nakazawa is registered with Nara Medical University as a postdoctoral fellow member paying registration fees.
