# Supplementary material for: An efficient feeder-free and chemically-defined expansion strategy for highly purified natural killer cells derived from human cord blood
Source: Regen Ther. 2023 Jun 1;24:32–42. doi: 10.1016/j.reth.2023.05.006 (PMC10247952; doi:10.1016/j.reth.2023.05.006)
Supplement: Multimedia component 1 [file mmc1.docx]

# Supplementary materials and methods

Following antibodies were used in this study.

| Antibody name | Supplier | Identifying number | Clone | Conjugated dye |
| --- | --- | --- | --- | --- |
| APC conjugated Mouse anti-Human CD56 (NCAM-1) | BD | 555518 | B159 | APC |
| FITC conjugated Mouse Anti human CD56 (NCAM-1) | BD | 562794 | B159 | FITC |
| PE conjugated Mouse anti-Rab, NHP, Dog, Hu CD11a (Integrin α L chain, LFA-1) | BD | 555384 | HI111 | PE |
| PE Mouse Anti-Human NKG2D (CD314) | BD | 557940 | 1D1 | PE |
| PE conjugated Mouse anti-Human DNAM-1 (CD226) | BD | 559789 | DX11 | PE |
| PE Mouse anti-Human NKp30 (CD337) | Beckman Coulter | IM3709 | Z25 | PE |
| PE Mouse anti-Human NKp44 (CD336) | Beckman Coulter | IM3710 | Z231 | PE |
| PE conjugated Mouse anti-Human NKp46 (CD335) | Beckman Coulter | IM3711 | BAB281 | PE |
| PE conjugated Mouse anti-Human CD159a (NKG2A) | Beckman Coulter | IM3291U | Z199 | PE |
| PE conjugated Mouse anti-NKB1 (KIR) | BD | 340484 | DX9 | PE |
| CD279 (PD-1)-APC | BioLegend | 329907 | EH12.2H7 | APC |
| CD223 (LAG-3)-APC, human | Miltenyi Biotech | 130-119-666 | REA351 | APC |
| Anti-TIM-3-APC, human | Miltenyi Biotech | 130-120-770 | E38-2E2 | APC |
| Anti-TIGIT-APC, human | Miltenyi Biotech | 130-116-935 | REA1004 | APC |
| CD96 (TACTILE)-APC, human | Miltenyi Biotech | 130-118-476 | REA195 | APC |
